# Self-Management Strategies for Low Back Pain Among Horticulture Workers: Protocol for a Type II Hybrid Effectiveness-Implementation Study

**DOI:** 10.2196/64817

**Published:** 2025-01-28

**Authors:** Kim Dunleavy, Heidi Liss Radunovich, Jason M Beneciuk, Boyi Hu, Yang Yang, Janeen McCormick Blythe, Kelly K Gurka

**Affiliations:** 1 Department of Physical Therapy College of Public Health and Health Professions University of Florida Gainesville, FL United States; 2 Department of Family, Youth and Community Sciences Institute of Food and Agricultural Sciences University of Florida Gainesville, FL United States; 3 Clinical Research Center Brooks Rehabilitation Jacksonville, FL United States; 4 Industrial & Systems Engineering Herbert Wertheim College of Engineering University of Florida Gainesville, FL United States; 5 Department of Statistics Franklin College of Arts and Sciences University of Georgia Athens, GA United States; 6 Department of Pediatrics School of Medicine University of Virginia Charlottesville, VA United States; 7 Department of Epidemiology College of Public Health and Health Professions University of Florida Gainesville, FL United States

**Keywords:** low back pain, self-management, implementation, horticulture workers, video training, video feedback, text message reminders, agriculture, ergonomic, nonpharmacological

## Abstract

**Background:**

Low back pain (LBP) is highly prevalent and disabling, especially in agriculture sectors. However, there is a gap in LBP prevention and intervention studies in these physically demanding occupations, and to date, no studies have focused on horticulture workers. Given the challenges of implementing interventions for those working in small businesses, self-management offers an attractive and feasible option to address work-related risk factors and manage LBP.

**Objective:**

This study will (1) investigate the effectiveness of self-management strategies for nursery and landscape workers by comparing within-subject control and intervention periods and (2) determine if adoption and effectiveness differs between participants randomly assigned to review self-management videos only and those who also receive multimodal implementation support. We will also identify contextual factors impacting effectiveness and implementation.

**Methods:**

A pragmatic, mixed methods, hybrid effectiveness and implementation design will be used to compare back pain with work tasks, disability, medication and substance use, and psychological factors between a baseline control and intervention periods. We aim to recruit 122 English- and Spanish-speaking horticulture workers with back pain, 30 supervisors, and 12 focus group participants. Participants will review short video modules designed to increase awareness of opioid risk and introduce self-management and ergonomic choices and use 1 self-management and 1 ergonomic strategy for 10 weeks. They will be randomly assigned to 2 implementation groups: video modules only or video + multimodal personalized support (checklist guidance, review of video feedback for ergonomic problem-solving, and text message reminders). Questionnaires will be administered at 3-month time points: baseline, pre- and postintervention, and at 3 and 6 months. Qualitative analysis of field notes, open-ended comments, and focus groups will expand understanding of results with comprehensive documentation of the context, barriers and facilitators, and reasons for adoption.

**Results:**

The project was funded on September 29, 2023 (Centers for Disease Control and Prevention National Institute of Occupational Health and Safety, CDC NIOSH; U54OH011230-07S1), as a core research grant for the Southeast Coastal Center for Agricultural Health and Safety. The design, creation, and editing of English and Spanish videos was completed in June 2024 after comprehensive formative evaluation. Enrollment began in June 2024 with anticipated completion in 2027.

**Conclusions:**

We hypothesize that both self-management interventions will result in reductions in work task pain and disability and that the video enhanced with multimodal personalized support will result in greater reductions than the video alone. If self-management is effective, mitigating pain positively impacts quality of life, productivity, and retention, while increasing the use of nonpharmacological alternatives to opioids addresses an important public health issue. Implementation aims will help inform reasons for results, barriers and facilitators, and potential for similar interventions in these and similar industries with physically challenging outdoor work.

**Trial Registration:**

ClinicalTrials.gov NCT06153199; http://clinicaltrials.gov/study/NCT06153199

**International Registered Report Identifier (IRRID):**

DERR1-10.2196/64817

## Introduction

### Background

Low back pain (LBP) is the most common musculoskeletal disorder among agricultural and horticultural workers [1-5], negatively impacting worker’s physical and mental health, increasing absenteeism, and shortening work longevity [6-11]. Further, LBP contributes to lower productivity, loss of experienced workers, and increased workman’s compensation costs. The financial impact of high out-of-pocket costs and lack of paid leave often drive agricultural and horticulture workers to seek pain relief from prescription medications and illicit substances [12-14]. In one study of farmers with LBP, 71% relied on pain medication without consulting a physician compared to only 35% of white-collar workers [15].

Viable alternatives to pharmacological management of LBP have never been more important [16,17]. Despite guidelines recommending nonpharmacologic management, opioids are commonly prescribed for work-related injury and pain more frequently than for nonoccupational musculoskeletal disorders [18-22]. Although opioid prescribing has declined gradually since 2010, opioid fatalities from prescription and illicit synthetic opioids have continued to rise [20,23]. The precipitous increase in opioid overdose associated with fentanyl manufactured in clandestine laboratories [23,24] has elevated the importance of highlighting and promoting uptake of nonpharmacological alternatives for management of LBP [20]. In rural areas, opioid prescription rates are particularly high [25], and access to nonpharmacologic treatment options is limited [16]. A preliminary study supporting this protocol found that over half of agricultural workers (n=129) reported using opioids at some point [14]. Of those who had used opioids, most (77%) reported they received a prescription for a work-related injury. Participants explicitly linked work hazards and chronic pain with increased risk of opioid dependence, citing the need to continue working and “live with pain” as a cultural norm contributing to the problem [14]. While progression to opioid use disorder is complex, opioid misuse has devastating effects on health and work. A systematic review of the impact of prescription medications for occupational musculoskeletal injuries found that opioid use was associated with slower return to work, longer duration of disability, poorer self-reported work function, and poorer functional improvements [26]. Opioid prescriptions within the first 2 weeks of a work-related injury, opioid use for longer than 7 days, and higher dose supplies are associated with subsequent work disability or delayed recovery and greater work loss [26]. Owners of nursery and landscaping businesses in Florida reported problems with turnover, frequent absences, and low productivity among workers who used opioids, resulting in decreased labor availability [14]. Landscape and nursery work characteristics including low skill discretion, job strain (low control and high demand), high physical effort, and heavy lifting are risk factors for opioid use disorder [27]. Few opioid prevention efforts have been focused on underlying work-related factors such as musculoskeletal pain [18,28]. The substantial worker and employer burden of LBP in the horticulture industry and the devastating effects of opioid dependency highlight the need for efforts to manage and limit LBP and reduce unwarranted opioid use for musculoskeletal injury. Strategies to (1) increase workers’ awareness of the risks and consequences of opioid use, (2) encourage workers to discuss options with health care providers after more acute injuries, and (3) provide alternatives for nonpharmacological strategies to manage pain or limit ergonomic stress [28] may increase knowledge and self-efficacy while reducing stigma [20,28].

Self-management is defined as the ability to manage pain, treatment, psychosocial, and lifestyle implications of a chronic condition [29,30], with the overall goal for individuals to safely manage symptoms and lifestyle without seeking unnecessary medical intervention [30-33]. Encouraging active involvement and empowering individuals to select options to manage work-related pain tailored to each worker’s circumstances and preferences encompasses the principles of self-management for chronic conditions [6]. Effective self-management strategies should optimize pain coping strategies to manage and reduce symptom exacerbation through problem-solving [30]. Self-management is recommended for the management of chronic LBP in a number of guidelines and strategic initiatives [16,34,35], with moderate effectiveness for pain, and small to moderate effectiveness for pain-related disability reported in a systematic review and meta-analysis [36]. Increasing self-management of chronic pain that frequently limits work activities is one of the Health People 2030’s targets [35]. However, most studies investigating effectiveness of self-management approaches have involved patients receiving clinical care rather than groups who continue to work in physically demanding occupations. Self-management skills such as problem-solving, action planning, self-tailoring, and self-monitoring may provide a worker-centered approach for horticulture workers to promote self-efficacy and coping skills for adjusting work activities and managing pain [29,30,37].

There are limited training initiatives to address musculoskeletal pain for horticulture workers and most workers rely on supervisor’s advice or learning by experience. Many studies have concluded that education can positively influence work-related musculoskeletal pain by promoting behavioral change, modifying health beliefs, and improving attitudes, although gaps in optimal implementation persist [38]. Although some studies reported additive effects of improving physical activity or ergonomic adjustments [38], none focused on agriculture, nursery, or landscape workers. Collectively, there is a crucial need to determine optimal implementation of effective self-management programs for workers, especially for those who do not have resources to receive treatment in typical health care settings [39,40]. Therefore, examining the effectiveness of training to assist workers with learning self-management and ergonomic strategies will provide valuable information, while studying implementation outcomes will help determine engagement, adoption, and feasibility.

### Prior Work

This protocol builds from a preliminary study of streamlined individualized participatory ergonomic (PE) methods in clam aquaculture workers with similar physically challenging work [39,41]. Workers with chronic LBP were introduced to ergonomic concepts to reduce risk, selected 3 pertinent and acceptable strategies from a list, reviewed videos, and received text reminders. Significant improvements in disability, work-task pain, pain anxiety, and coping compared to baseline were observed (*P*<.05; n=19), with pain improvements greater than published minimal detectable change for 74% of the participants. The methods were both feasible and acceptable. These promising results led to recommendations to establish whether these approaches are effective in a larger cohort over longer timeframes, in different contexts, and with follow up [39].

We will investigate the effectiveness of self-management and ergonomic individual choices along with behavior change support for nursery and landscape workers. The first aim is to determine if self-management videos combined with multimodal personalized support is more effective than self-management videos alone for improving LBP management among horticulture workers. We hypothesize that compared to the video modules alone, the combined multimodal personalized support will result in (1) greater reductions in work task pain and disability, (2) lower prevalence of high-impact chronic pain, (3) greater reduction in substance use to manage pain, (4) greater reeducation in pain anxiety and depression, and (5) improved coping and self-efficacy. The second aim is to identify contextual factors that impact engagement, adoption, effectiveness, and implementation of nonopioid alternatives for LBP self-management. Understanding the external context and individual and team characteristics will help explain the results, provide important implementation perspectives, and inform translation.

## Methods

### Study Design

The exploration and preparation phases of the study were started in 2023, with the video training module creation, evaluation, modifications, and production completed in June 2024. A 2-arm pragmatic randomized controlled hybrid type II effectiveness and implementation study [42-44] will be conducted using mixed methods and a within-subject control period. The Standards for Reporting Implementation Studies (StaRI) standards checklist is included in Multimedia Appendix 1.

### Ethical Considerations

The study was approved by the University of Florida Institutional Review Board (IRB202300756) and registered as a clinical trial through ClinicalTrials.gov (NCT06153199). Participants will be compensated for their time. Workers will receive US $25 for the baseline data collection and US $50 for subsequent steps for a possible total of US $225, while supervisors or owners will receive US $50 for the 2 data collection sessions for a possible total of US $100.

Employers will provide permission to discuss the study with workers and to conduct the study in the workplace. After determining eligibility, research staff will describe the purpose of the study, study-related procedures, risks, and benefits, and participants will sign an informed consent if they agree to participate. We will ensure that participants understand that participation is voluntary and that they can withdraw from the study at any time without consequences. A Certificate of Confidentiality (42 USC §241(d)) will protect participant privacy related to potentially sensitive information about the use of pain medication and other substances. If individual workers are not eligible or do not want to participate, they will be able to view the educational videos with their teams. If participants who are randomly assigned to the intervention enhanced with multimodal support are not comfortable being videotaped, we will provide deidentified video clips of other workers with blurred faces performing tasks similar to the tasks they have identified as difficult due to pain.

### Recruitment

#### Recruitment Methods

We will recruit nursery and landscape workers in Florida through community partners and contacts from the Southeastern Coastal Center for Agricultural Health and Safety. We will use direct communication, email lists, websites, word-of-mouth referrals, and community meeting announcements to identify employers who are interested, followed by presenting the study to workers. We will recruit workers with a range of disability, pain impact, and persistence, anticipating that participants will be primarily male, with higher numbers of female participants in the nursery teams, and that approximately 50% will identify Spanish as their first language. Initial contact and permission will be through owners and supervisors to enroll nursery and landscape businesses and their employees.

#### Inclusion and Exclusion Criteria

Criteria for inclusion are (1) working full time (30 hours or more per week) in nursery or landscape businesses, (2) 18 years of age or older, (3) English or Spanish speaking, and (4) experiencing continuous or intermittent LBP over the past 3 months. We will exclude individuals who (1) report history of trauma, major spine surgery, or spinal nerve blocks in the past year; (2) are seeking disability or workmen's compensation, or (3) self-disclose pregnancy.

#### Sample Size Projections

A priori sample size was calculated using data from the pilot study comparing pain and disability measures pre and post–self-management PE interventions for 19 seafood workers [39]. For each participant and pain or disability outcome, averages of 4 baseline measurements and 2 postintervention measurements were used to calculate the difference in pre- to postintervention outcomes. Mean changes over a control period are assumed to have a mean 0 difference but share the same SD as the intervention period. Changes over the 2 periods for the same person are assumed to be independent. Data were simulated from a simple linear mixed model (LMM):

*Y_ijk_* = α*_i_* + β_1_*X_ijk_Z_ijk_* + β_2_(1 – *X_ijk_*)*Z_ijk_* + ε*_ijk_*

In this model equation, *Y_ijk_* is the outcome of observation period *k* of individual *j* in cluster *i*, *X_ijk_* indicates the intervention group (1: video + support, 0: video), *Z_ijk_* indicates the intervention period (1: intervention period, 0: baseline period), α*_i_* is the random intercept of cluster *i*, and ε*_ijk_* is the error term. The variance of α*_i_* was set as 5% of ε*_ijk_*, such that the intraclass correlation is close to 0.05. For each set of simulated data, a generalized estimation equation model was used to test the null hypothesis β_1_=0. We ran 1000 simulations to calculate the power for each of the different settings of the number of clusters and cluster sizes. Table 1 shows the power based on individual randomization. Fitting a LMM yielded comparable results. The planned enrollment of 102 participants (24 clusters) will provide adequate power for the primary outcomes, pain with most difficult work activities and total disability. An additional 20% enrollment for attrition results in a total anticipated sample of 30 clusters and 122 participants (teams of 3-5 participants) for the comparative effectiveness goals.

**Table 1:** Statistical power (%) based on individual randomization for differentiating between the 2 implementation groups using preliminary data.

Number of clusters	Cluster size	Projected Sample size	Power (%) calculated using results from Dunleavy et al. [39].			
			Pain with most difficult work activities (mean –16.1, SD 18.8)^a^	Average pain (mean –5.2, SD 17.3)^a^	Total disability (mean –2.5, SD 3.5)^a^	Difficulty level with most problematic work activity (mean 9.0, SD 28.5)^a^
**20**						
	3	60	96.9	28.4	88.7	33.8
	5	100	99.8	43.9	98.3	49.5
	10	200	100	73.5	100	73.5
**25**						
	3	75	98.9	32.3	93.3	40.7
	5	125	100	48.6	99.4	57.5
	10	250	100	80	100	84
**30**						
	3	90	99.5	38.2	97.1	44.3
	5	150	100	56.3	99.8	64.4
	10	300	100	87.3	100	88.8

^a^Mean difference, SD from Dunleavy et al [39].

#### Target Enrollment and Randomization

Our target enrollment of 122 workers will be recruited over 3 years starting in June 2024 with anticipated completion in 2027. For each team, we will enroll at least 1 supervisor or owner to provide support and comments on the overall feasibility and results, for 30 supervisors. In year 4, we will also invite 12 stakeholders to participate in focus groups (see aim 2) for a total expected enrollment of 164. Participants will be randomized to one the intervention groups after the baseline control period using stratified block randomization by type of work (nursery, landscape), and within each employer group to ensure a 1:1 distribution. The random assignment table was created prospectively by the statistician and participant numbers are generated in the REDCap (Research Electronic Data Capture) software (version 9.1.1; Vanderbilt University) [45] when baseline data are downloaded to reduce the risk of bias. Participants will receive incentives after completion of the surveys, training, and interviews.

### Data Collection

#### Timelines

**Figure 1:**
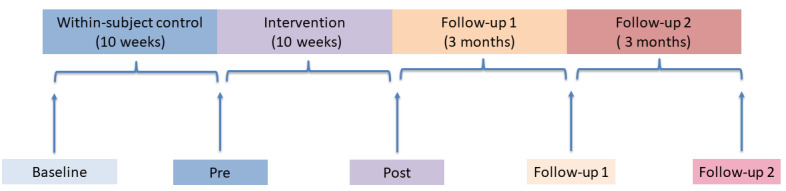
Timelines.

Five measurement periods will allow longitudinal comparison of change from the within-subject control period (10 weeks), across the pre- and postintervention period (10 weeks), and follow-up (3 and 6 months; Figure 1).

#### Questionnaires

Questionnaires in English or Spanish will be administered in the workplace on tablets using the REDCap platform [45]. The REDCap is a secure, web-based software platform with questionnaire support and data management functions [45]. Primary dependent variables will be collected at all timepoints—pain (severity, interference, and persistence); pain with specific work tasks; disability; work ability; and pain medication use [46-51]. Affective or cognitive characteristics impacting adoption and effectiveness (secondary dependent variables or potential confounders) such as coping, fear, anxiety, and depression will also be collected [52-55]. Branching logic will be used to pipe answers for the most difficult work activities and choices of strategies into subsequent questionnaires. In addition to demographics, medical and work characteristics will be collected. Disability will be assessed using Roland-Morris Disability Index [49], Patient Specific Functional Index (PSFS) for work tasks considered most difficult due to back pain [39,50,56-58], and Work Ability Index instruments [48]. Pain constructs from the National Pain Strategy recommendations (severity, interference, persistence, and impact) [46,47], along with pain with the specific work tasks identified in the PSFS, will be measured using Likert scales [39]. Substance use will be recorded using the National Institute on Drug Abuse quick screen [23] with additional questions on over-the-counter pain medication and other substances. Affective and cognitive measures include the Pain Anxiety Symptom Scale [53], Center for Epidemiological Studies Depression Scale short form [52], Coping Strategy Questionnaire [55,59,60], and Self-Efficacy for Chronic Condition management short form [54]. Instruments were translated by a certified translation agency if validated Spanish versions were not available.

#### Qualitative Methods

**Figure 2:**
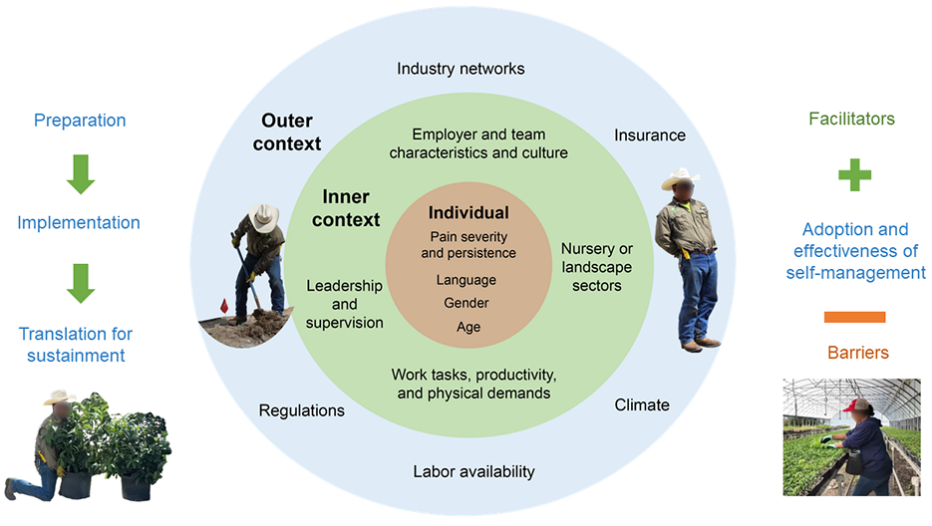
Contextual framework: selected components from the EPIS (Exploration, Preparation, Implementation, Sustainment) Model.

**Figure 3:**
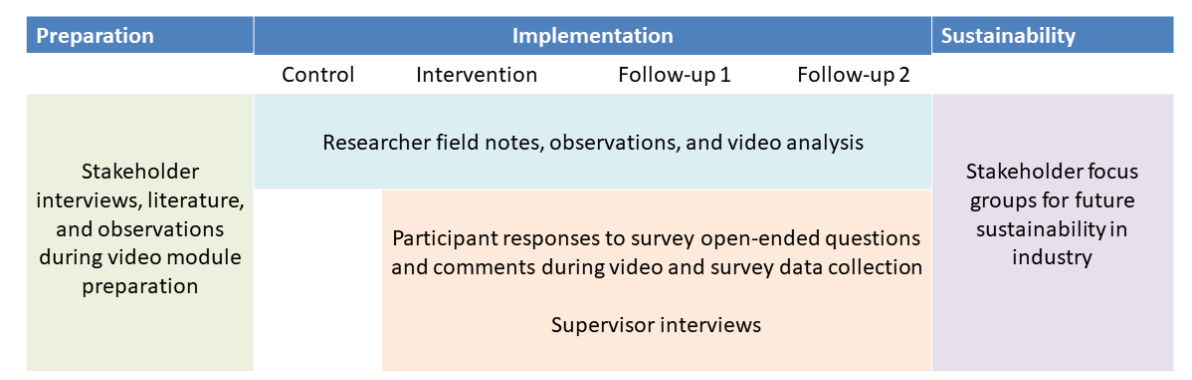
Qualitative methods.

Understanding the context (circumstances and unique factors surrounding the study) will help explain results and provide implementation outcomes [43,61,62]. We will use relevant components of the Exploration, Preparation, Implementation, Sustainment conceptual framework, including inner (individual and team) and outer (industry, regulations, and environment) contextual factors (Figure 2) [63]. Qualitative data will be compiled from (1) researcher observations and field notes; (2) anchor-based and open-ended questions reflecting engagement (opinions of intervention delivery and support, recommendations to others), adoption (use of strategies in past 7 days), and feasibility (ease of use, barriers and facilitators); (3) perceived effectiveness (including global rating of change); (4) supervisor and worker comments; and (5) data collected during final stakeholder focus groups (Figure 3). Spanish-speaking research team members will contribute to observations and if needed, will translate comments. We will record comments, expansions, and clarifications and will ask workers specifically about the acceptability of introducing information about opioid risk and self-management at home and in the workplace. Supervisors and owners will be asked to provide their opinions from an organizational perspective. Recommendations will be confirmed, prioritized, and expanded ensuring robust and iterative analysis.

In addition, 2-3 focus groups will be conducted during the last quarter of the study. Industry representatives, employers, supervisors, and select participants will be invited to contribute their opinions. We will invite industry leaders committed to support wide-spread dissemination and translation of interventions, and workers able to comment on their real-world perspectives with 1 focus group conducted in Spanish. Focus group questions will help explain findings and patterns identified throughout the study and generate recommendations for future interventions. Question prompts will reflect any similarities or differences generated from earlier data analysis to confirm themes and conclusions, further ensuring triangulation and rigor. Interview and focus group recordings will be recorded using participant numbers only, transcribed, and translated if appropriate [64]. Comprehensive data analysis will be conducted progressively throughout the study, contributing to triangulation and trustworthiness of the qualitative analysis [64,65].

#### Risk Exposure for Tasks

Participants will be videotaped while performing their self-identified most difficult work tasks at baseline and after the intervention. After categorizing video clips by tasks, subtasks, and variations using hierarchical task analysis, the risk for each task will be categorized using the Rapid Entire Body Assessment (REBA) checklist [59,60]. The REBA is a commonly used measure to reflect ergonomic risk in the workplace and reflects extreme postures in tree nursery workers better than other observational instruments [66]. We will use a software system (Tumeke) [67], which identifies and reports the REBA for the postures and movements in the cycle with the highest risk to help with consistency of analysis. The risk assessment measures will be documented for specific tasks rather than used to compare individual change due to the variability of tasks and potential difficulty capturing the exact same tasks for all participants. Tasks will be recorded as changed or unchanged.

To further understand the impact of work adjustments and variations, simulations of various ergonomic scenarios will be conducted in laboratory settings with 3D analysis. Tasks with multiple variations and those which are changed will be selected and replicated. These simulations will involve creating different work environments and task setups to replicate the typical conditions encountered by the participants. By analyzing these simulated scenarios, we aim to identify potential ergonomic improvements and assess relative risk for variations of the tasks rather than evaluating individual change.

### Intervention

#### Video Training Module Design and Preparation

Three short video modules were created specifically for nursery and landscape workers, tasks, and environments. The instructional design was informed by stakeholder discussions, review of current evidence, targeted in-depth interviews with industry experts (insurance safety consultants, association leadership, owners, supervisors, and workers; n=15), and work observation conducted during the preparation phase. Stakeholders also completed surveys which helped prioritize a preliminary list of work tasks considered most problematic for back pain.

Nursery and landscape worker characteristics informed design of multimedia visual representation of work activities and content with representation of genders and ethnicities in the videos and resource materials. Scripts were designed and evaluated for language consistent with lower than eighth grade literacy level to meet the lowest anticipated educational level, while structure and organization to reduce cognitive load, and design accounting for multiple language versions was built in from the first draft (Textbox 1).

**Figure 4:**
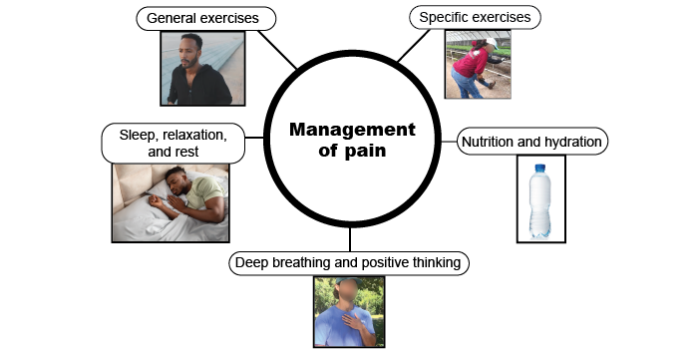
Self-management strategy options.

**Figure 5:**
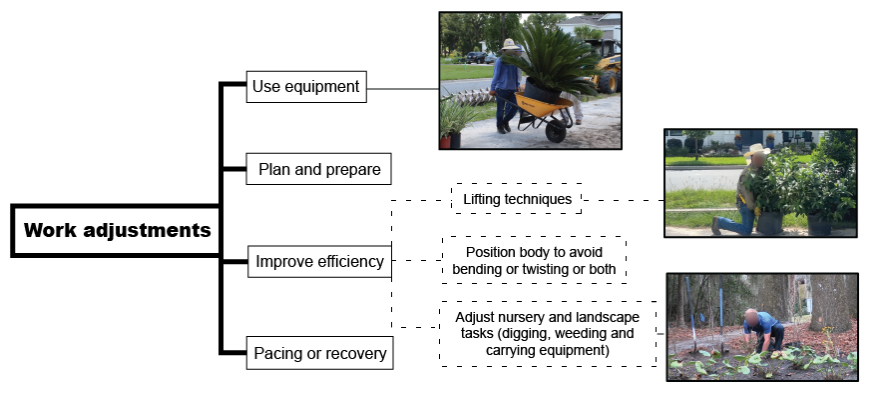
Ergonomic strategy options.

The videos emphasize the importance of managing and limiting pain using active nonpharmacological self-management and conserving work longevity to facilitate ongoing support of family. The modules cover (1) the need to manage pain without relying on medication and substances, as well as the risk of opioid use; (2) self-management strategies for pain at home and at work (general exercise, specific back exercise, nutrition and hydration, deep breathing and positive thinking, rest, relaxation, and sleep); and (3) work-specific ergonomic strategies to address physical risk factors during nursery and landscape tasks. The videos encourage selection of relevant strategies for pain management of exacerbations, emphasize prevention through lifestyle and behaviors such as exercise, nutrition, and sleep, and proactively planning work tasks and adjusting movement to minimize risk (Figures 4 and 5). Sector-specific video clips of relevant work activities were filmed in nursery and landscape work settings and represent workers with a mix of genders and ethnicities, along with key stakeholder commentary to provide relevance.

**Design choices matched to characteristics.**

**Multimedia visual representation**

- Videos and narration; minimal use of text with simple language, short sentences, images to represent key concepts, and advance organizers [68,69].
- Check list support materials (print) [68,69].
- Infographics and resource materials.
- Text messages include graphics and gifs for movement.

**Health literacy and cognitive load considerations**

- Literacy levels lower than eighth grade [68,70].
- Organization to maximize working memory and reduce cognitive load using advance organizers, smaller “chunks” and grouping of concepts [71].
- Graphics to represent choices as advance organizers.
- Short modules to minimize overload.
- Encouragement to choose relevant options for self-efficacy.
- Message design and visual representation, for example, focus on positive options for movement and work adjustments rather than what not to do or representation using symbols with negative connotations such as red crosses.
- Contrasting recommended movement in color with those that should be adjusted in black and white video.
- Music and screen transitions to divide concepts.

**Multiple languages**

- English version design and initial formative evaluation to identify and adjust vocabulary and grammar difficult to translate into Spanish.
- Length of clips adjusted to time needed for Spanish narration using transitions.

**Feasibility for workplace**

- Video delivered on iPads in 5-minute modules to maintain attention to use in the field for “toolbox” talks, team discussions, or as part of general safety meetings.
- Options based on the work and team location rather than requiring classroom settings to promote feasibility.

#### Video Formative Evaluation

The design incorporated iterative feedback from instructional design and health literacy experts and input from the research team who have expertise in pain management, rehabilitation, psychology, opioid misuse, and ergonomics. The scripts were evaluated for health literacy and inclusivity, followed by formative review by the experts in our multidisciplinary team. The adjusted English script was evaluated by owners, supervisors, workers, communication and industry experts, as well as bilingual stakeholders for words and concepts that are difficult to translate.

Supervisors, workers, and outside stakeholders (n=27) reviewed the video for content, format, representation, understanding, and provided comments. Additional video clips were added, and some content reorganized for flow and organization to reduce cognitive load. Final narration was completed after the second revision. The script was translated into Spanish by a certified agency and reviewed again for consistency, language, messaging, and cultural acceptability, prior to adding the Spanish narration and titles. Spanish speaking supervisors, workers, and stakeholders (n=12) also evaluated the Spanish videos with minor changes.

#### Self-Management and Ergonomic Options and Implementation Strategies

The self-management and ergonomic options (Figures 4 and 5) and implementation strategies (Table 2) were selected using evidence, stakeholder interviews, and established theoretical frameworks (health beliefs [72,73], self-determination [74-76], social cognitive [77,78], and Bronfenbrenner’s Ecological Systems Theories [79]). The theoretical frameworks will help to interpret effectiveness outcomes for the group and for different subgroup characteristics and determine if interventions are modifiable to fit with internal and external contexts [80].

After the within-subject control period, participants will be randomly assigned to 1 of the 2 implementation arms. Workers in both groups will view the tailored educational video modules in the workplace and select 1 self-management (Figure 4), and 1 ergonomic strategy (Figure 5), to use during the 10-week intervention period. Workers assigned to the arm enhanced with additional support (video + support) group will be provided checklists to use while reviewing the videos and asked to consider strategies they do not use or could use more frequently [81]. To assist workers with problem-solving, participants in this group will be shown video recordings of their own work and encouraged to problem-solve ergonomic alternatives [39,41,81]. Finally, the workers in the video + support group will receive weekly SMS text message reminders to support their specific choices. These automated messages will be delivered using the Mosio text platform [82] at the beginning (ergonomics) and at the end (self-management) of the week. The messages are preprogrammed and will include graphics and gifs to represent concepts from the videos. Supervisors will also be asked to support workers for ergonomic choices and provide their opinions of engagement, adoption, and feasibility.

**Table 2:** Implementation outcome measures.

Implementation strategy	Engagement	Adoption	Feasibility
Tailored short educational video modules delivered in the workplace using iPads	Acceptability, relevance—questions during or after training Opinions of the videos and delivery	Number of ergonomic and self-management strategies used Frequency of strategy use Intent to use strategies in the future	Ease of use of strategy Facilitators or barriers Time for delivery Feasibility in the workplace environment
Worker choice of preferred strategies matched to self-identified most difficult work tasks	Acceptability	Number of ergonomic and self-management strategies used Frequency of strategy use Intent to use strategies in the future	Ease of use Barriers and facilitators
Supervisor involvement	Supervisor engagement (observation)	Uptake and involvement of workers from supervisor’s perspectives	Supervisor interviews—feasibility, reasons for adoption, fit with organization, and environment Barriers and facilitators
Visual feedback for positions and movements	Qualitative comments and questions during review of video	Use of strategies	Time Recognition of positions and postures Opinions of relevance and acceptability
Ergonomic problem-solving	Involvement in problem-solving Supervisor comment discussion among workers	Use of strategies	Opinions of ergonomic strategies and problem-solving Supervisor observations of suggestions for work tasks, changes in work practices
Text message reminders including images and gifs	Questions and return texts		Feasibility for workers Opinions of text messages

### Data Analysis

#### Aim 1

LMMs or generalized linear mixed models (GLMM) will be used to compare within-subject changes in the primary and secondary outcomes (1) over time (baseline, pre, and post) between the intervention periods and the control period and (2) between video and video + support interventions, depending on the distributional feature of the outcome. A general regression structure will be used:

*g*(*EY_ijk_*) = α*_i_* + *γ_j_* + *β’X_ijk_*

In this equation, *Y_ijk_* is the change of the outcome over observation period *k* of individual *j* in cluster *i*; *g*(*EY_ijk_*) is the link function for the mean of *Y_ijk_*; α*_i_* and *γ_j_* are random intercepts specific to cluster *i* and individual *j*, respectively; and *X_ijk_* and *β* are vectors of covariates and associated coefficients. *X_ijk_* indicates intervention (1: intervention video+support, 0: control video only), and baseline characteristics (demographics, occupation, and baseline pain or disability levels). For continuous outcomes that are symmetrically distributed (after transformation), the following traditional LMM will be used:

*Y_ijk_* = α*_i_* + *γ_j_* + *β’X_ijk_* + ε*_ijk_*

where ε*_ijk_* are normal random errors. For skewed continuous outcomes, a GLMM with a gamma distribution will be used. Intervention effects will be represented by mean differences in the continuous outcome between the comparison groups. For categorical outcomes such as prevalence of chronic high impact pain, we will either dichotomize the outcome and fit a logistic mixed model or directly fit a Bayesian multinomial logit model that is available in the R package. Intervention effects will be represented by odds ratios. We will also reparameterize the model to obtain relative risks for measuring intervention effectiveness. These models will account for correlations within each team, as well as within each subject. Results will be verified by general estimating equation models in case within-team or within-subject correlation structures are mis-specified. Missing values for either outcome or covariates will be managed by multiple imputation or the Expectation-Maximization algorithm.

#### Aim 2

Qualitative analysis will help explain effectiveness and implementation outcomes concurrently [43,61]. Contextual factors that support or hinder engagement, adoption (use of interventions), feasibility, and effectiveness will be described. We will examine general barriers and facilitators and specific patterns for workers in the nursery and landscape sectors. The qualitative analysis will be used to develop a comprehensive matrix of team context, organization, and individual characteristics to explain effectiveness of interventions and implementation results. Using directed deductive content analysis, initial codes will be developed from analysis of preparatory interviews for (1) contextual factors; 2) barriers and facilitators contributing to the degree of impact of the interventions; and (3) opinions of ease of use, overall satisfaction, and reasons for engagement, adoption, and feasibility results. We will use NVivo qualitative analysis software (version 14; Lumivero) to code content and identify categories and subcategories to describe patterns and relationships that emerge. We anticipate comparing themes for (1) intervention groups, (2) participants with different degrees of pain severity and persistence, (3) individuals and teams who adopt and implement the strategies consistently compared to those who do not, (4) individuals who respond positively to the interventions compared to those who do not improve, and (5) subsectors of the industry. Once themes are identified, interconnections, interactions, and relationships will be described and explained. Recommendations for future implementation and sustainability will be confirmed and prioritized.

## Results

Preparatory literature searches, stakeholder interviews, and observations were started in January 2023 and completed in August 2023. The stakeholder interviews confirmed the limited formal training for prevention of musculoskeletal repetitive strain injury and overall need for addressing LBP in the industry. The project was funded on September 29, 2023 (CDC NIOSH U54OH011230-07S1), as a core research project for the Southeast Coastal Center for Agricultural Health and Safety. The video recording and creation started in April 2023 and was completed in June 2024. Formative evaluation of drafts 1 and 2 of the videos, revisions, translation into Spanish, and narration were completed in April 2024, and final video edits were completed in June 2024. The first group was enrolled in June 2024. As of December 6, 2024, we have enrolled 14 participants and 4 supervisors from 3 employers. Data collection is anticipated to be completed in late 2027 or early 2028 with plans for final analysis and dissemination of findings shortly thereafter. Qualitative data for Aim 2 will be coded and analyzed progressively for team characteristics and trends.

## Discussion

### Importance of Study Findings

In this paper, we present the protocol for a randomized within-subject controlled hybrid intervention study with coprimary aims to examine the effectiveness of self-management videos combined with multimodal personalized support compared to self-management videos alone for improving LBP and disability in nursery and landscape workers, as well as to identify contextual factors that impact implementation including engagement, adoption, effectiveness, and feasibility. Improving self-management strategies to reduce transition from intermittent to high-impact chronic LBP will contribute to important Healthy People 2030 objectives [35] while addressing research and practice gaps to mitigate musculoskeletal pain.

In nursery and landscape businesses, prevention of musculoskeletal injuries has not typically received high priority. Despite the size and growth of the industry, horticulture has rarely been included with other agriculture sectors in studies addressing musculoskeletal injury prevalence [5] or risk factors [5,6]. There is a death of intervention studies to prevent or mitigate LBP in agriculture [2,10,66], with few intervention studies specifically targeting nursery and landscape populations [5]. Feasible, easily implemented interventions to manage pain and reduce risk factors are essential for small businesses in the horticulture industry who face major challenges with labor availability. Variability in work environments, seasonal fluctuation in workloads, weather constraints, and productivity requirements make the adaptability, efficiency, and ease of adoption paramount for any training intervention. Current “training on the-job” from more experienced workers and supervisors is challenging with turnover and difficulty retaining experience workers who can pass on best practices. Efficient delivery methods providing structured evidence-based information and strategies would provide consistency. Short videos tailored for both sectors are a pragmatic option to address the needs while remaining flexible for work priorities and productivity. This study will contribute to knowledge about the effectiveness of interventions specifically tailored to the characteristics, work tasks, and work settings for these workers.

Awareness of the risks and consequences will highlight the importance of nonopioid alternatives [14]. Opioid education is not effectively integrated into workplace safety training but embedding discussion of opioid risk within a more holistic approach to prevention and mitigation of LBP may promote help-seeking behavior and reduce stigma and communication barriers [20]. Introducing the risk of opioids along with options to manage pain using self-management and ergonomic alternatives is likely to be received more positively than opioid education alone.

Awkward postures, repetitive actions, and intermittent lifting, digging, or shoveling have been identified as inherent ergonomic risk factors for landscape workers [5]. Similar risks are present in nursery work with awkward stooped trunk postures, repetitive low load lifting, and intermittent heavy lifting [10,11]. Larger companies often have more access to mechanical equipment, while small work units seldom have sufficient resources for major engineering redesign recommended in the NIOSH hierarchy of controls [6]. Modifying the way people work or reinforcing the use of available equipment in small businesses is, therefore, more realistic than major equipment modifications. Typically, smaller companies do not have access to ergonomic and safety experts, and may not provide health care benefits. Feasible, easily implemented interventions to manage pain and reduce risk factors are, therefore, needed for businesses in the nursery and landscape industry.

PE approaches involve workers in identifying possible solutions to reduce work-related stress and have been used in a variety of ways in large manufacturing, construction, and health care settings [83-86]. The evidence for effectiveness of PE in large corporations is mixed for group changes for time off work, return to work, self-assessed productivity, and prevalence of LBP [85,87-94]. In contrast, PE has been found to improve overall perceived health, decrease back pain intensity, and reduce absences due to back pain [94]. Comprehensive PE interventions are often time-consuming and challenging to implement across large corporations. Solutions suggested by workers may be feasible, but changing organizational processes can be difficult. Thus, PE has been suggested to be especially beneficial for small teams, enterprises with restricted finances, and rural or less developed areas [86,95,96]. Although PE has not been studied extensively in agricultural settings [39,41,86,95,96], there is some evidence of lowered ergonomic risk and improved management of symptoms among farmers [86]. In another study, construction workers reported significantly less general fatigue after a typical workday, and increased influence on their work compared to controls [81]. A modified approach to encourage workers to choose relevant options for changing their work activities to minimize ergonomic stresses may be feasible to empower nursery and landscape workers to manage back pain [40].

In this study, changes in the primary (pain, disability, and medication use) and secondary (pain-related anxiety, coping, self-efficacy) measures from the preintervention to postintervention timepoints will be compared to a within-subject baseline within-subject period. We will also compare 2 forms of training implementation—video modules alone and video enhanced with multimodal personalized support. We hypothesize that the video + support will facilitate greater engagement, and increased adoption, thereby contributing to greater reductions in work task pain and disability compared to a baseline control period than the video alone. If either intervention is effective, mitigating pain and enhancing knowledge, skills, and attitudes to manage pain successfully is likely to impact quality of life, productivity, and retention. Improvements in pain and disability could result in decreased use of pain medication and other substances, important outcome public health priorities. Even if observed differences are not significant, identifying characteristics of those who respond will contribute evidence for preventing the transition from low to high-impact chronic pain. The mixed methods will help explain results and surrounding inner and outer contextual factors, facilitators and barriers contributing to implementation success for individuals, teams, and specific strategy choices [44]. Qualitative analysis will also inform recommendations and provide ideas for sustainability of effective interventions. Analysis from multiple perspectives will also help explain emerging or complex factors inherent with the dynamic workplace environment and business models, to guide decisions for others considering these approaches. If self-management is effective for horticulture workers (or specific subgroups), simultaneously studying implementation outcomes (engagement, adoption, and feasibility) will facilitate translation of the intervention from research to practice and scaling to the workforce. Potential limitations that may impact the success of this study are consistent with other hybrid effectiveness implementation studies. First, although we anticipate that our recruitment goals are achievable, external factors such as productivity requirements may impact owner and supervisor willingness to allow workers to participate. As attrition is expected, we plan to enroll additional participants to achieve adequate power. We will emphasize benefits for employers such as limiting absenteeism, improving retention, and reducing potential risks of medication or substance use to manage pain while working. Second, we also anticipate that owners and supervisors who agree to participate are already committed to supporting workers. This may result in a sample of workers associated with positive contextual factors that may not be generalizable to all employers. However, these factors will be documented to help interpret results from an implementation perspective. Third, weather and seasonal variations are likely to impact productivity and, therefore, willingness to participate, particularly for landscape companies. These factors will also impact scheduling for data collection, for example, hurricane or weather delays. We have built in a 2-week window for data collection to accommodate the scheduling but there may be a need for additional flexibility. Finally, cultural, language, and educational literacy variations are expected. All instruments and materials have been translated and checked thoroughly while research assistants who speak Spanish fluently are essential.

### Conclusions

Interventions to reduce the burden of LBP that consider the unique characteristics and business models within the nursery and landscape sectors are lacking. Given the high incidence of LBP, the risk of opioid use, impact on labor availability and productivity, and the limited attention to musculoskeletal disorders in these sectors, this study will provide valuable information to address both employer and worker needs. The hybrid type II effectiveness and implementation study will investigate the effectiveness of self-management interventions for pain and disability, along with the engagement, adoption, and feasibility, for 2 forms of implementation while also documenting the context, facilitators, and barriers for nursery and landscape businesses [44]. Increased awareness of opioid risk and options for alternate nonpharmacological strategies to manage pain will also provide an important contribution to a high priority public health challenge. We anticipate self-management will result in reduced pain and disability. Further, targeting self-management can empower workers and improve pain coping, reducing pain anxiety, depression, and associated use of pain medications. Short video training modules presenting realistic strategies to manage pain and limit work stress are likely to be feasible in the workplace while multimodal personalized support is likely to facilitate engagement and adoption of context-specific solutions. The qualitative methods used will help elucidate whether these options are feasible for small- to medium-sized horticulture businesses, document barriers and facilitators, and describe the inner and outer contextual factors influencing adoption and effectiveness. If the video and multimodal personalized support intervention is more effective than viewing training modules alone, this knowledge will inform best practices and may be easily adapted for other physically challenging agricultural sectors.

## Appendix

Multimedia Appendix 1 STARI (Standards for Reporting Implementation Studies) checklist.

## Abbreviations

CDC: Centers for Disease Control and Prevention
GLMM: generalized linear mixed model
LBP: low back pain
LMM: linear mixed model
NIOSH: National Institute of Occupational Health and Safety
PE: participatory ergonomic
PSFS: Patient Specific Functional Index
REBA: Rapid Entire Body Assessment
REDCap: Research Electronic Data Capture
StaRI: Standards for Reporting Implementation Studies

## Data Availability

Deidentified data will be made available to researchers upon written request. A data-sharing agreement will be developed and signed that provides commitments to (1) use the data only for research purposes and not to identify any individual; (2) securing the data using appropriate computer technology and security protocols; and (3) destroying or returning the data after analyses have been completed. This data sharing plan may be modified as recommended by the funding agency or university policies at the time of study completion.
